# PPCS: A Progressive Popularity-Aware Caching Scheme for Edge-Based Cache Redundancy Avoidance in Information-Centric Networks

**DOI:** 10.3390/s19030694

**Published:** 2019-02-08

**Authors:** Quang Ngoc Nguyen, Jiang Liu, Zhenni Pan, Ilias Benkacem, Toshitaka Tsuda, Tarik Taleb, Shigeru Shimamoto, Takuro Sato

**Affiliations:** 1 Department of Communications and Computer Engineering, Faculty of Science and Engineering, Waseda University, Shinjuku-ku, Tokyo 169-0051, Japan; tsuda-toshitaka@nifty.com (T.T.); shima@waseda.jp (S.S.); t-sato@waseda.jp (T.S.); 2 Global Center for Science and Engineering, Faculty of Science and Engineering, Waseda University, Shinjuku-ku, Tokyo 169-0051, Japan; liujiang@aoni.waseda.jp (J.L.); zhenni.pan@aoni.waseda.jp (Z.P.); 3 Department of Communications and Networking, Aalto University, 02150 Espoo, Finland; ilias.benkacem@aalto.fi (I.B.); tarik.taleb@aalto.fi (T.T.); 4 Computer and Information Security Department, Sejong University, Seoul 143-747, South Korea; 5 Center for Wireless Communications; University of Oulu, FI-90014 Oulu, Finland

**Keywords:** information-centric networking (ICN), cache redundancy, caching policies, cache optimization, edge computing, named-data networking (NDN), Future Internet (FI), network design

## Abstract

This article proposes a novel chunk-based caching scheme known as the Progressive Popularity-Aware Caching Scheme (PPCS) to improve content availability and eliminate the cache redundancy issue of Information-Centric Networking (ICN). Particularly, the proposal considers both entire-object caching and partial-progressive caching for popular and non-popular content objects, respectively. In the case that the content is not popular enough, PPCS first caches initial chunks of the content at the edge node and then progressively continues caching subsequent chunks at upstream Content Nodes (CNs) along the delivery path over time, according to the content popularity and each CN position. Therefore, PPCS efficiently avoids wasting cache space for storing on-path content duplicates and improves cache diversity by allowing no more than one replica of a specified content to be cached. To enable a complete ICN caching solution for communication networks, we also propose an autonomous replacement policy to optimize the cache utilization by maximizing the utility of each CN from caching content items. By simulation, we show that PPCS, utilizing edge-computing for the joint optimization of caching decision and replacement policies, considerably outperforms relevant existing ICN caching strategies in terms of latency (number of hops), cache redundancy, and content availability (hit rate), especially when the CN’s cache size is small.

## 1. Introduction

Information-Centric Networking (ICN) has been proposed by V. Jacobson et al. [1] with built-in key features of in-network caching and naming scheme for the Future Internet (FI) [2]. Given that we are coping with the explosive growth of demand traffic for the Big Data era with increasing content size in which global Internet data traffic in 2019 is estimated to be 64 times as of 2005 [3], ICN is becoming one of the most promising FI designs. The reason is that the ICN mechanism provides great benefits over the current IP-based Internet architecture including content dissemination, security, mobility support, and scalability.

The in-network caching capability, however, also brings new challenges, especially the implementation and cache utilization concerns. The reason is that, by default, ICN wastes cache space to store the same objects at all Content Nodes (CNs) along the path regardless of content popularity. In fact, even though caching is a core research topic due to its critical role for ICN performance, the cache redundancy problem is still largely unexplored since most of the existing ICN caching schemes store every piece of data (content chunks) in the CNs. This mechanism requires a large cache storage capacity and then causes highly redundant and inefficient cache utilization within ICN interconnections. The redundancy issue of ICN even gets more challenging in the current Big Data era due to the fact that a popular content with large size like HD (High Definition) video usually receives a huge number of access times from users. Then, it results in large network traffic and cache redundancy in ICN. In addition, the caching performance is related to CN cache size and the available cache resource is limited, compared to all available content objects (content catalog). Thus, it is necessary to address this theme and minimize the redundancy level in ICN for a large-scale and practical ICN deployment.

Motivated by these facts, the presented paper proposes a novel cache management scheme, namely Progressive Popularity-Aware Caching Scheme (PPCS), including both content placement and replacement policies to minimize cache redundancy, reduce network traffic, and provide efficient content delivery to users. Specifically, to improve cache diversity and eliminate cache redundancy, PPCS introduces an intelligent caching scheme by calculating the number of chunks (per each object) to be cached at each CN for the downstream delivery process. From this, PPCS dynamically adjusts the window cache size, according to the content popularity and CN position. We also propose a utility-based cache replacement strategy instead of using the Least Recently Used (LRU) and the default eviction policy of ICN to improve its cache utilization. By shifting our focus to edge-based cache redundancy avoidance for efficient and feasible ICN communications, this paper then differs from our previous work in ICN [4,5,6] in which we mainly integrated Green networking into ICN for our optimized energy-efficient ICN model.

The contribution of the proposal is three-fold as follows:

(1). We propose a hybrid strategy of both partial and whole data caching, according to the content popularity to minimize the on-path caching redundancy, given that the popularity level is defined, according to the content request arrival rate and network data traffic. Utilizing the merits of edge computing, this mechanism ensures that there is, at most, one copy of content stored along the path from the Content Provider (CP) to the user;
(2). The autonomous replacement policy is performed at each CN to prevent unimportant content items from being cached in ICN, especially at edge nodes, for the efficient cache utilization of limited cache resources in ICN;
(3). The joint optimization of caching decision and replacement policy improves the overall ICN system performance by pushing content chunks closer to the user and differentiating the caching policies of popular content objects from that of unpopular items.

Overall, this article contributes to the new concept of cache redundancy avoidance and efficient content dissemination in ICN with innovative ideas of popularity-aware caching policies as well as the utility-based replacement strategy. This concept is leveraged by an edge-based implementation utilizing key features of ICN to minimize cache redundancy and the server hit rate at the same time. Specifically, PPCS realizes a chunk-based popularity-aware caching scheme, which lets content be cached initially at edge nodes and lets the caching move upward to the core network gradually, as the content popularity diminishes. Thus, the more popular content objects are expected to be at the edge nodes (close to users) while the less/non-popular content objects are cached on content nodes near the CP. Such a combined solution ensures the high cache hit rate and low latency for end-users by pushing content data closer to them, especially in the case of highly popular content objects. The evaluation results show that PPCS realizes a highly scalable ICN approach with beneficial content distribution, which matches the rapid growths of content demands and wireless users in the era of the Internet of Things (IoT). The proposal also enables a practical ICN approach, given that ICN endures a relatively high cache redundancy by caching the whole data of all the newly arrived content through its default-caching scheme.

The formal existing ICN documentations of major standardization bodies like ITU and IETF have not explicitly addressed the cache redundancy problem in ICN. Therefore, this research aims to build a highly efficient and scalable caching scheme, which eliminates unnecessary content duplicates and enhances the feasibility of ICN infrastructure deployment for FI. This proposal then acts as a practical and potential approach, which matches the design goals of future networks [7] and achieves the target of the “Connect 2020” program [8] toward establishing global-scale and widely-applicable ubiquitous networks.

## 2. Related Work

The appearance of ICN [1,9] has brought us a new Internet design with embedded security, built-in mobility support, and a higher network performance and lower latency compared to the current host-to-host model. In ICN, caching scheme is an active research topic because the default caching decision, Leave Copy Everywhere (LCE), is relatively inefficient and endures a poor caching performance. In particular, LCE treats all content in the same way and unnecessarily caches the same content items at all CNs along the path. It then results in low content diversity, high resource consumption, and great cache redundancy.

Regarding the notable work in ICN caching, I. Psaras et al. proposed ProbCache [10], which cooperatively caches the content objects at CNs, according to the hop reduction to CP (distance from the server) and cache capacity of Content Routers (CRs) along the transmission path. In Leave Copy Down (LCD) [11], the content object is stored at the neighbor downstream CN along the path when a cache hit happens. In this way, LCD pushes a replica of content one-hop-closer to the user whenever a content request is successfully served. However, this may take a longer time for the user to get content with a small number of cache hits and also leave redundancy in ICN. The authors in Reference [12] proposed Age-Based cooperative Caching (ABC) in ICN by introducing the content age for expiration. A CR stores a content longer if it is more popular and/or closer to the edge side. In Reference [13], the Time Aware Least Recently Used (TLRU) was presented as cache management in ICN with a lifetime span aware eviction scheme extended from LRU. Y. Thomas et al. proposed Object-Oriented Packet Caching (OPC) to study and improve the impact of packet-caching in ICN [14]. In Reference [15], the authors proposed a hierarchical caching with different cache durations as a method for advertising cached content in the control plane. In our prior work, we designed an integrated caching and routing solution to maximize the chance of finding nearby cached content for efficient cache utilization in ICN [16]. In Reference [17], the authors applied the concept of centrality to Content-Centric Networking (CCN, a typical ICN platform) for the goal of realizing a centrality-measure-based caching scheme in ICN. An analytical content distribution framework for popular content in ICN was developed in Reference [18]. The authors proposed a non-cooperative game-theoretic approach with Nash caching strategies in ICN interconnections with transit and access ICNs and a CP. They also consider pricing policy for popular content in such an ICN system under the assumption that caching cost in ICN is inversely proportional to the content popularity. Y. Zhang et al. proposed a cache replacement scheme known as the Popularity Prediction Caching (PPC) for chunk-level video caching by investigating the relationship of video chunks and user watching behavior [19]. Specifically, PPC caches the predicted future with the most popular content in place of the least predicted popular ones and outperforms the widely used replacement policies in ICN including popularity-based strategy, LRU, LFU (Least Frequently Use), and FIFO (First In First Out). In Reference [20], the authors proposed an interesting chunking method for partial caching with a theoretical analysis in ICN. They presented the idea of optimal chunking instead of caching the complete content items in ICN. However, the chunk size is too small and it is not clear how to obtain the optimal number of chunks and each chunk size, in the case of the optimal heterogeneous chunking. To avoid the high complexity, they also proposed the homogenous chunking with an equally sized chunk, namely HECTIC (HighEst Cost Item Caching), which spreads content probability using the utility-based chunks along the transmission path. This method outperforms the alternative performance, but the evaluation results on tree topology are limited with only 4000 content objects and the file size is about 10 MB.

Another approach for optimizing content placement in ICN, it is pre-fetching content items in advance, as identified in Reference [21]. In this context, we also presented a caching scheme utilizing proactive-caching to improve the cache performance of our Green ICN model [22,23]. Thanks to the smart scheduler and the ‘fake’ Interest mechanism, the proposed system performed well, but it was limited to the context of Intelligent Transport System (ITS). The proactive/pre-fetching content method requires additional bandwidth consumption.

These studies improved the caching performance, but they did not directly consider the cache redundancy issue and usually cache the entire content object at multiple CNs along the path. Given that partial caching has been largely unexplored in ICN, besides Reference [20], the most related study to this paper is WAVE [24], which also prevents cache redundancy via chunk-level data transmission. WAVE stores content chunks at the core router at first and then gradually populates the content toward the edge side based on content popularity. As the WAVE initially caches content chunks at the core-side, it causes higher latency due to the underutilization of edge CNs. In contrast, this research focuses on caching at the edge side due to its impact on cache performance, given that edge computing can realize the practical case study in IoT [25]. Moreover, we consider the entire-object caching if the content is popular enough and propose an autonomous eviction policy to further optimize the efficiency of overall cache utilization.

## 3. The Needs and Merits of an Edge-Based ICN Processing Framework

Different from the existing host-centric networks, ICN realizes a flexible data plane for efficient data dissemination. Particularly, ICN enables user-driven applications by including built-in mobility support. ICN also enables distributed collaborative processing at the edge and intermediate content nodes to reduce the dependency on the original content source or the cloud, as a practical solution to realize quick responses with low latency. In this way, data can be exchanged between end-users/devices and edge or intermediate fog nodes (along the path from the edge and the core network) with respective data, instead of heavily depending on the connection to the cloud/content provider, which causes high traffic load, and a congestion ratio at the core network and the cloud content provider.

As a result, edge computing will leverage the benefits of ICN to realize the efficient content-centric communications by serving a substantial amount of content data at the edge-side or the fog nodes located near the content requesters. These ICN nodes then act as a proxy in an Autonomous System (AS), which can store content and serve data directly to end users efficiently, thanks to the ICN in-network caching feature and unique name-based communications. In addition, in ICN, the requests for the same content are aggregated at content routers to facilitate the congestion control. Accordingly, ICN natively and fully supports the flexible edge-based communications by consolidating vertical data-driven services between users and content nodes (fog and edge ICN nodes) via its smart data discovery (via nearby cached content at ICN nodes), name-based routing, and in-network processing mechanisms. Moreover, ICN provides content-based security, rather than communication channel security as of the IP-based network, which is very sensitive to a\Denial of Service (DoS) attacks from the malicious attackers. Particularly, different from IP-based networks where dedicated communication channel between two IP addresses of two hosts is maintained and secured, ICN aims to assure the integrity of content by securing content itself (i.e., built-in security measure). In addition, the recent and notable work show that fog computing-based content aware in ICN can be used for security services in social networks [26]. Edge-based in-memory caching can save energy in the multi-tier heterogeneous network [27] and edge-centric computing together with CCN is beneficial for orchestration in the 5G Radio Access Network (RAN) [28].

To reflect the need for an edge-based ICN processing framework, in this study, we propose PPCS as an edge-based cache redundancy avoidance in ICN. For this research, we select the Name Data Networking (NDN) platform [29] among various ICN-based frameworks as a practical, incremental overlay deployment approach since it has the backward-compatible capability with IP [2,29]. The reason is that it is difficult to substitute the whole Global IP address in the existing Internet architecture. Then, the clean-slate ICN-based approaches are undesirable and hardly deployable for FI in reality. In addition, a hierarchical naming structure in NDN enables packet aggregation and network scalability. A content in NDN is split into chunks, and a chunk is the data unit for content transmission. NDN uses the content name to address, forward and retrieve data through two types of packet. The Interest packet addresses the content name and is forwarded to a valid content source. If there is a cache hit for the requested content, the Data packet returns the data to the user along the reversed path. In addition, each CN in NDN has three fundamental data structures: Content Store (CS) as the cache space for caching content items, together with the Pending Interest Table (PIT) and the Forwarding Information Base (FIB) for packet forwarding. Our recent work in NDN include: utilizing Artificial Intelligence (AI) to realize an intelligent classification framework for the quality of service (QoS) performance improvement in ICN [30], the leveraging Network Functions Virtualization (NFV), and Multi-Access Edge Computing (MEC) technologies by integrating ICN into CDN (Content Delivery Network) for an efficient content delivery service, which avoids congestion in the core network side [31] and designs a smart congestion control mechanism for the green IoT sensor-enabled ICN system [32]. Note that, even though we focus on NDN, this proposal is not limited to NDN and can be applied to any other ICN framework.

## 4. Progressive Popularity-Based Caching Scheme

This section presents the detailed system model and design of PPCS, which eliminates cache redundancy and optimizes cache performance in ICN. In general, PPCS avoids caching the same object twice along the path and objects are cached either entirely or partially from the edge side to CP with respect to content popularity. Moreover, we propose a smart replacement policy, which selectively picks up the most recent and popular contents at each CN to enhance the cache utilization.

### 4.1. System Model

**Figure 1:**
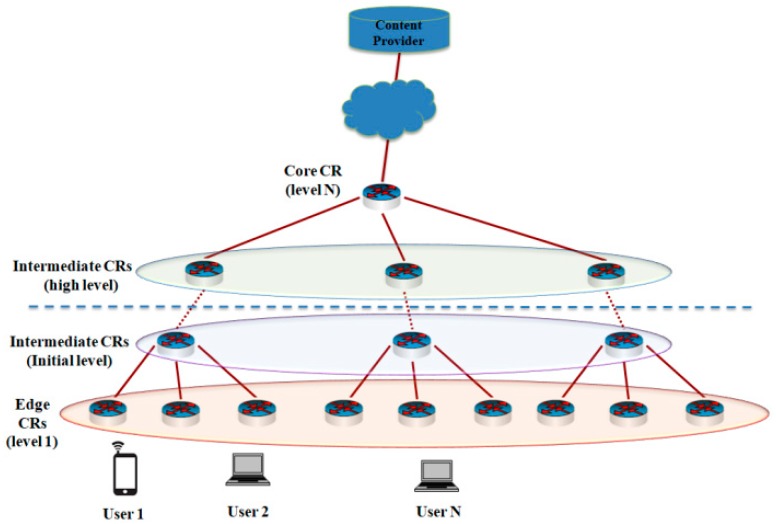
Hierarchical network topology for PPCS.

For network topology, we consider the general hierarchical network topology because it represents a commonly-used model for research work in ICN in References [33,34]. In the ICN interconnections, CP acts as the root node, which stores all the available content items and edge CRs refer to the leaves of network topology. CP attaches to the core CR (level N) while edge CRs, i.e., leaf nodes at level 1, are connected to users (Figure 1). The core CR can be considered as the gateway, which connects to the local repository or remote CPs. Each CN employs a CS to cache the content chunks, and all CRs are assumed to have the same cache capacity (CS size).

Content is divided into fixed-size chunks and they are transmitted and cached at CRs sequentially along the content delivery path based on chunk Identifier (Chunk ID). Particularly, the Chunk ID is identified by the concatenation of the content file name and the sequence number of the chunk.

Users generate and send the Interest packets to corresponding CR to ask for desired content objects. If the content cannot be served locally (cache miss), it will be forwarded to the next higher-level CR along the path in ICN interconnections. The workload and network traffic then reflect the characteristics of CRs at different levels. Only the less popular content items with cache misses (specified by low content request frequency) are forwarded to higher level CNs whereas the most popular content objects are expected to be at the edge side since they are replicated more frequently compared to non-popular ones. Thus, to maximize the number of Interest packets, it can be served locally, i.e., higher cache diversity. In order to prevent cache redundancy, we aim to push content chunks closer to the user, and especially spread the popular content objects to the network edge. The detailed proposal will be clarified in the next parts of this section.

Note that, in this hierarchical network design, a lower level node can connect to multiple nodes at a higher level in which the transmission path is selected based on a minimum number of hop counts. Therefore, if one intermediate node has some issue like link corruptions and congestion. Then another content node at the same level can be selected to establish the newly available delivery path. This strategy then enhances the feasibility of ICN for future network deployment as in ICN. The requested content data can be accessed from a replica instead of the only content source such as in the current IP-based Internet architecture. In this type of network interconnections, ICN realizes a more flexible forwarding scheme via its in-network caching implementation by deploying extensive cache structure and provides content security. Sending malicious request packets to a host then becomes difficult because ICN cares only about content not hosts. In addition, for flow control, NDN only returns, at most, one Data packet for an Interest packet to control flow and reduce the congestion rate.

In addition, note that, even though we mentioned the content users connect to edge routers and send the content requests there, the edge routers can also directly connect with IoT devices, e.g., sensors to collect, transmit, and process data to realize a smart-sensing design in the same way as we did in our recent study on smart IoT sensor-enabled ICN system [32]. In addition, for convenience, we use the terms node and router (CN and CR) as well as the server and CP interchangeably.

### 4.2. Popularity-Based Caching Decision

#### 4.2.1. PPCS’s Processing Algorithms for Interest and Data Packets

Since the edge cache can account for a large portion of content requests from users [35], PPCS protects against on-path content duplicates utilizing both the entire object and partial caching. Particularly, PPCS caches less popular content chunks at CRs on the path from the edge to core CR while all data or most chunks of more popular content are cached at the edge CRs (near users). This hybrid mechanism is efficient because users may only express Interests for a portion of the file, rather than the whole data, especially in the case of large-size multimedia files. For instance, about 80% of YouTube videos are interrupted before their duration due to the lack of user interest [36]. In addition, the foremost chunks of content are likely to be accessed more frequently than others [3]. Therefore, it is more efficient to cache the foremost chunks of unpopular content first at edge CR than cache the whole data (limited cache space). Note that the content popularity is classified according to the content popularity distribution model and the data traffic to the edge side.

For this goal, we define *count_c_* as the total number of requests for a specified content *c* sent to the edge node on the path. Specifically, *count_c_* is a cumulative value, which records the total number of consumer requests in the (past) fixed period. By counting the content access frequency of the object at the edge node, *count_c_* acts as a measure for the dynamic popularity of content over time, which corresponds to the number of requests from users for the content. Let *c_t_* be the threshold value of content access frequency. Then if *count_c_* is equal or greater than *c_t_*, the content is a popular content. Otherwise, it is an unpopular content. Note that the value of *c_t_* is not strictly fixed and can be changed during different periods because the relative content popularity of a certain content can change over time, according to the Interest arrival rate within a period. We then denote $n_{k}^{c}$. as the number of consecutive chunks of a content *c* to be cached at level-k CR along the path. Specifically, to reflect the sequential nature of successive content chunks’ accesses, the starting chunk ID of $n_{k}^{c}$. follows the last chunk stored at a lower level CR, i.e., the downstream CN at level k-1 (along the retrieval path). Furthermore, let *n_c_* be the total number of chunks of content *c*.

From this, we design a hybrid caching decision strategy of both entire content caching and partial caching to place content in ICN, according to the content popularity level strategically. To achieve this, we define *CB_c_* (Caching Bit) as a binary variable to check whether an arriving content c is a popular content or not. If *CB_c_* is equal to one (*CB_c_* = 1), PPCS decides to cache the whole data of the (popular) content items at the edge node. This method is reasonable because, as observed, the total number of times that an important/popular content is accessed, which may exceed the combined number from a huge set of unpopular ones.

For unpopular content items, the proposal enables a flexible segmentation strategy that adaptively adjusts the number of chunks to be cached (caching window) based on the value of *count_c_* (number of content requests at edge CN). To ensure that no content duplication appears along the path from the edge node to core CR, PPCS initially accumulates only a small fraction of unpopular content items at the edge side by caching foremost chunks at edge CR. Then, in case that the number of content accesses at the edge side increases over time, the progressive caching mechanism uses an exponentially increasing function to grow the number of chunks (per each content) to be cached at each CN sequentially along the path from the edge Content Routers (CRs) to core CRs. By adjusting the cache window size at CRs along the path, PPCS quickly spreads the content chunks to reflect the increased content popularity. This policy is repeated until the content is promoted to be a first-class content and becomes a popular item (i.e., *count_c_* is equal to or greater than the threshold value *c_t_*). At that time, the content data is cached at the edge node (Algorithm 1). The proposed progressive and incremental caching scheme then stores sufficient initial chunks of unpopular content at CNs along the path to diminish the latency and match the user access pattern.


**Algorithm 1. Interest packet processing Algorithm**
  //cache at edge routers  **if**
*c* is a new content (i.e., did not get request before) **then**    *count_c_* = 1    $n_{1}^{c}\;=\;\lceil \frac{1}{N}\;n_{c}\rceil$  **else**    *count_c_* = *count_c_* + 1    **if** (CB_c_ == 1) **then**      **if** (1 < *count_c_* < c_t_) **then**        $n_{1}^{c}\;=\;\lceil \frac{count_{c}}{c_{t}}\;n_{c}\rceil$      **else**        **if** (*count_c_* ≥ c_t_) **then**          $n_{1}^{c}$ = n_c_          CB_c_ = 0        **end if**      **end if**    **end if**  **end if**  //cache at higher level CRs along the delivery path  **if** (CB_c_ == 1) **then**    **for** k = 2 to N **do**      $n_{k}^{c}\;=\;\lceil n_{1}^{c}\;\cdot \;m^{\left(1-k\right)}\rceil$//m is the exponential coefficient for content chunk caching      **if**
$(n_{1}+\;\sum_{j=2}^{k}n_{j}\;$> $n_{c}$) **then**        $n_{k}^{c}\;=\;n_{c}-(\sum_{j=2}^{k-1}n_{j}^{c}$ + $n_{1}^{c}$)      **end if**    **end for**    set cache field in PIT   **end if**

Regarding the chunk transmission process, we use the caching Flag as the indicator bit to mark whether the CR should cache the content chunks when the node forwards data to the user. In case CN will cache the chunks, then the Flag mark in the Data packet is cleared once the CN caches the marked chunks. Moreover, if the whole content can be fully retrieved from the edge node to an intermediate CN along the path, then all the cached chunks of content at higher level CNs will be deleted to save cache space at these CNs (Algorithm 2). This light collaborative caching policy is realized via the core node (involved in the delivery path), which acts as the centralized node utilizing the signaling packets implicitly.


**Algorithm 2. Data packet processing Algorithm**
  **if** content cache field is set in PIT entry **then**    cache data in CS    CB_c_ = 0    clear the cache Flag   **end if**  forward Data packet  **if** the entire content can be fully retrieved along the path by CNs from the edge CR to an intermediate CR at level k **then**    delete all cached chunks of the content at from level (k + 1) to core CR CNs  **end if**

The detailed algorithms of the Interest packet and Data packet processing of PPCS are shown in Algorithm 1; Algorithm 2, respectively.

Note that two or more small size unpopular content items may be as good as a popular large size content due to the accumulative popularity of all the content from the group of the small-sized content items. However, we consider that this method is more difficult to perform and not reasonable because, for the same caching memory, a large number of small-sized content refers to a high overhead from multiple packet/message exchanges. In addition, due to the nature of content popularity within a specified period, a small-size un-popular content is not likely to be asked again at the next period. Even when the unpopular content is asked again, partial or whole data retransmission from the closest node (with cached data) in ICN interconnections is acceptable since the number of packets is limited due to the content’s small size. Worse still, the content grouping policy may produce higher complexity and latency compared to PPCS. Even the number of Interest packets from the group of small-size content and a single popular content is comparable.

#### 4.2.2. A Case-Study of PPCS’s Caching Decision Strategies

**Figure 2:**
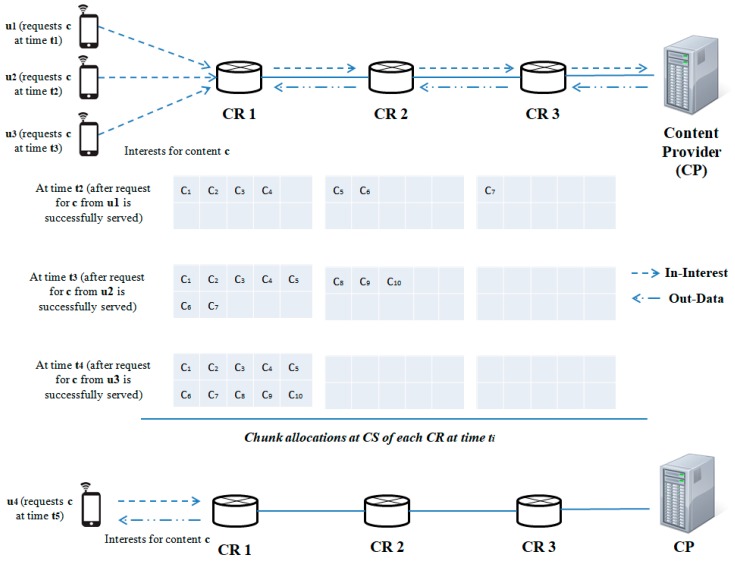
Use-case for caching decision strategies of PPCS.

To illustrate the operation of PPCS with these two algorithms, Figure 2 presents a simplified scenario with a network of three CNs and one CP. There are four users (*u_1_*, *u_2_*, *u_3_*, and *u_4_*) and they ask for the same content object *c* at a different time. User *u_1_* starts sending requests first and user *u_i+1_* begins sending a request for content *c* when user u_i_ successfully receives all the chunks of content *c*. Content c has a total of 10 chunks, i.e., *n_c_* = 10, and all CNs are also assumed to have the same cache size of 10 chunks, with the exponential coefficient for content chunk caching *m* = 2. Suppose that not all CRs stored any content chunks by the time user *u_1_* sends the content request for *c*. Therefore, when the file is first accessed, the first four chunks are marked for caching at the edge node (as $\left\lceil \frac{1}{3}10\right\rceil$ = 4). Following Algorithm 1, PPCS caches the next two chunks (*c_5_*, *c_6_*) at CR 2 and chunk *c_7_* is stored at CR 3. Assume that *c* has the threshold value *c_t_* = 3. Then, when user *u_2_* starts asking for contents, PPCS marks the first seven chunks and the last three chunks of *c* for caching at CR 1 and CR 2, respectively. Since the content can be fully retrieved by CR 1 and CR 2, the cached chunk at CR 3 (*c_7_*) is deleted (Algorithm 2). Lastly, when users *u_3_* and *u_4_* send interests for *c*, all the content chunks of *c* are stored at the edge node (CR 1). Users can get the content directly from CR 1. In addition, no chunk is stored at upstream CRs (CR 2 and CR 3) since *c* is promoted to be a popular content at time *t_4_* (i.e., *count_c_* ≥ *c_t_* when *u_3_* asks for *c*). Together, Algorithms 1 and 2 shape how much data traffic should flow along the delivery path for efficient and fast content dissemination through the ICN interconnections.

In short, PPCS accumulates content chunks from the edge side and then pushes the content chunks close to the user to reduce latency and network resource utilization considerably. By quickly populating the popular content objects to the edge network, the ICN system reduces the network load at the core side substantially because a huge number of requests for popular content can be served from edge CNs. For unpopular content items, partial caching corresponding to the dynamic access frequency of content is a promising approach because caching only a small portion of content can reduce latency efficiently [37]. This way is more beneficial for large-size (unpopular) content items since large size caching refers higher lookup overhead. In addition, in case of content with low popularity, caching initial chunks only requires a relatively small cache space at the edge side temporarily. Thus, for unpopular content items, only storing a fraction of data along the path (from the edge CN to the core side) enables a practical caching to improve cache diversity and content availability as well as diminish content cache redundancy at the same time.

### 4.3. Cache Replacement Policy

Although this article focuses on eliminating cache redundancy, to further optimize the cache utilization, we also propose a dynamic cache replacement, which optimizes the total utility of each CN over all cached content objects in CS through each cache replacement decision.

To decide which content should be discarded when the cache space of a CN is full, each content item in a CN is associated with a dynamic utility value that changes over time.

(1) $$u_{c}^{i}=\frac{f_{c}^{i}}{s_{c}},$$

where *s_c_*, $u_{c}^{i},$ and $f_{c}^{i}$ are the size of a certain content *c*, utility value, and access frequency of content *c* at a specified CN *i*, respectively. The higher value of utility states that the content has less chance to be discarded from CN because it is likely to be reused in the near future.

When a new distinct content comes to a CN, it initializes the utility value of the newly arrived content as well as updates the values of cached content objects. In this way, each CR checks and decides whether to replace a cached content with the new content for the most beneficial cache replacement, as follows:

- If the CN still has free cache space and the CR did not store the arriving content, then the CR will cache the new content item by caching content chunks sequentially until CS is full.
- When the CS is full, the chunks of cached content, which has the lowest utility value, will be the candidate to be dropped/discarded from the cache memory of CN. If there are multiple content items with the same value of utility, the LRU will be used to filter the least recently used content in CS. The CN then compares the utility of newly received content with the least-utility of cached content and begins to discard the content with a lower value for storing more beneficial content. This policy prevents popular content items from being replaced by less or unpopular ones.

Thus, the proposed replacement mechanism improves cache utilization of CNs by keeping the most active and popular content objects in cache space. The detailed algorithm of this utility-based cache replacement policy is shown in Algorithm 3.


**Algorithm 3. Content replacement policy of PPCS**
  **if** CS is not full **then**    cache content chunks (until CS is full)  **else**    **if** utility (c) ≥ min utility (c_i_) (i.e., minimum utility among all items cached in router i), **then**      replace cached content with minimum utility (c_i_) by the new distinct content c    **end if**  **end if**

To sum up, by calculating utility values of cached content items in CS and then comparing the lowest one with the utility of newly arrived content to each CN for the eviction decision in real-time, we realize a dynamic utility-based replacement policy, which can operate at CRs autonomously. Together with proposed caching decision at section III.B, PPCS makes the edge network realize highly popular objects and then dedicates more cache memory for storing more popular content at initial-levels CRs (edge side CRs). For the less popular ones, PPCS utilizes a progressive and popularity-proportional caching mechanism to accumulate content chunks along the path from the edge CRs to higher level CNs. This novel caching scheme then eliminates cache redundancy and makes the best use of cache memory to optimize cache performance in ICN. Note that, even though we propose PPCS in hierarchical networks, the proposal is definitely applicable to general ICN interconnections/topologies in which nodes at the initial levels refer to CRs closer to the user along the delivery path (edge side). CRs at higher levels are upstream and intermediate CRs along the path and core CRs are associated with the CP (root node). Specifically, for more complex topologies in which there are multiple routes from a specific edge node to the core CRs, the transmission path is selected as the route from the user’s attached edge node to an appropriate core node with the minimum hop-count. In this way, the network depth of each node is identified based on its connectivity with other nodes in ICN, and it is assigned to the lowest value in case the node has multiple distinguished connections.

## 5. Performance Evaluations and Discussion

In order to verify the efficiency of PPCS, we simulate the proposal and alternative strategies using ndnSIM [38], which is a widely-used ns-3 based NDN simulator. The network topology used for chunk-level simulation is five-level tree topology. The CP is connected to core CR at level four while the users connect to edge CRs at level one of the hierarchical network topology. The total number of objects (content catalogue size) is 100,000. Each CR has the three NDN fundamental data structures (CS, PIT, and FIB) and all the available content items are stored in CP. For simplicity, all content objects are assumed to have the same size of 400 MB (payload size of 1 KB), and each ‘parent’ node covers three ‘child’ nodes.

The links capacity between edge nodes and users is 100 Mbps and smaller than that of the links connecting CNs (1Gbps). A user follows the Poisson arrival process and requests three Interest packets per second to edge CNs. The content popularity is modelled by well-known Zipf distribution [39]. For the parameters used in the evaluation, we take values of alpha (skewness factor of Zipf distribution), m (exponential coefficient for content chunks caching), and CS (CR cache capacity) as 0.8, 2, and 50 full objects cache space for each CR if not otherwise specified. The key simulation parameters for the evaluations are summarized in Table 1.

We then use the same network environment and values of defined parameters to assess the proposal efficiency by comparing PPCS with five other relevant caching strategies in ICN (WAVE, HECTIC, LCD, ProbCache, and LCE). Note that the eviction policy in PPCS is based on the content utility of content objects in each CN (as stated in Section III.C). Similarly, HECTIC is a simple utility based caching strategy whereas other alternatives use LRU to replace the cached content with the longest non-access time when the CS of a CN is full. The following network metrics are evaluated.

**Figure 3:**
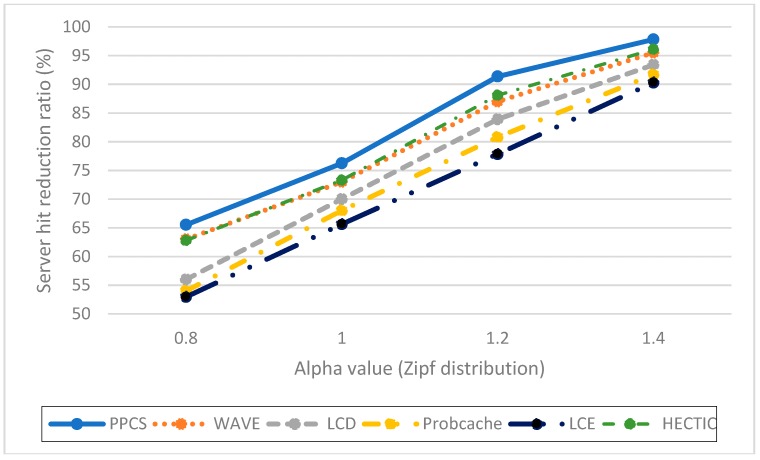
Server hit rate with various Alpha values.

**Figure 4:**
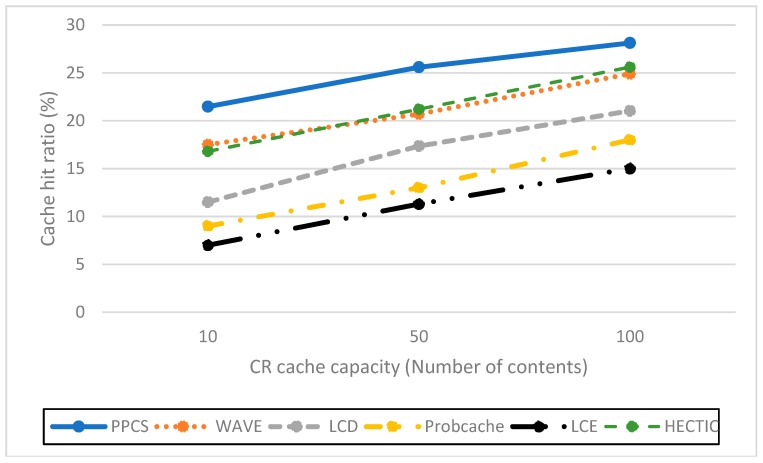
Cache hit rate corresponding to different CS sizes.

**Figure 5:**
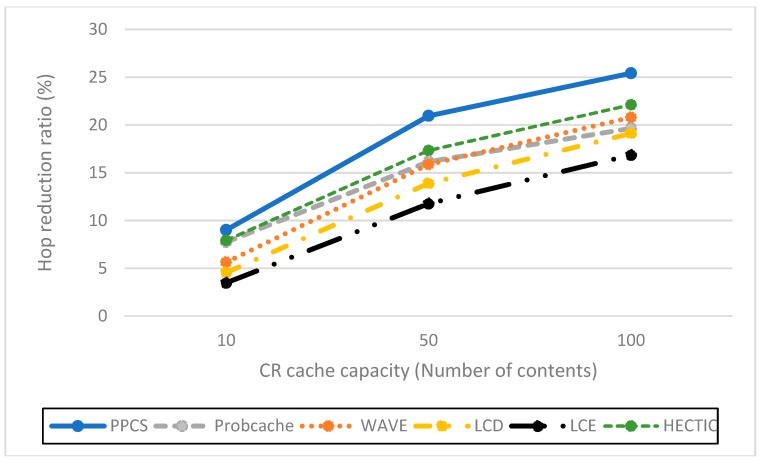
Hop reduction ratio versus different CS sizes.

**Figure 6:**
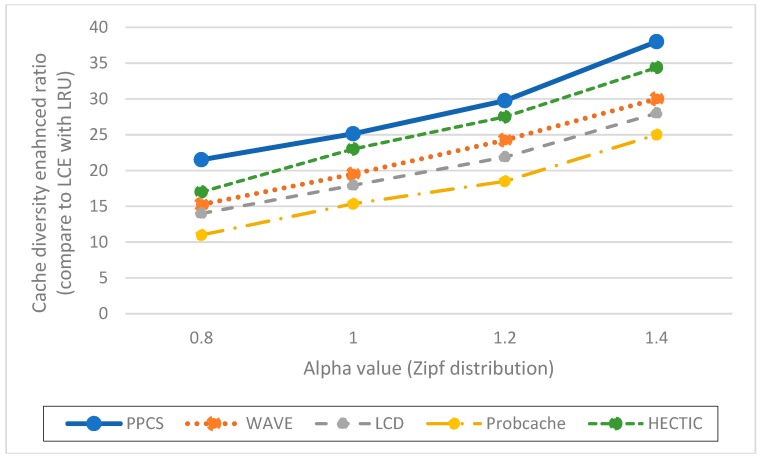
Cache diversity enhanced ratio corresponding to different Alpha values (compared to LCE with LRU).

- **Impact of the server hit reduction ratio over various Alpha values (Zipf distribution).** To point out the relation between caching performance and content popularity, we vary the value of Alpha from 0.8 to 1.4. When Alpha value increases, the popular contents are more dominant. Therefore, the server hit rates of all caching schemes reduce as expected. As shown in Figure 3, HECTIC, WAVE, and PPCS have good performance by enabling the partial caching policy to improve cache efficiency. In addition, PPCS has the lowest server hit rate because it realizes the highest level of content availability in ICN by enabling the integration of the entire and partial caching based on content popularity. We then demonstrate that PPCS can minimize the backbone bottleneck by diminishing traffic to the server substantially via the collaborative distributed processing at the edge and the intermediate content nodes along the delivery path in ICN. This result also verifies that, after a moderate number of chunks, partial caching produces insignificant benefit as stated in Reference [20], especially in the case of unpopular content items with a low-interest arrival rate.
- **Impact of the cache hit ratio with different CR cache capacities**. We abstract the cache size of each CN as the relative ratio to the content catalog (all available content) that varies from 10 to 100 content objects, which is equivalent to a fraction of 10^−4^ to 10^−3^ to the content catalog size. As expected, PPCS, HECTIC, and WAVE outperform other caching schemes since they do not waste cache space for storing redundant chunks in ICN, especially in the case of limited CN cache size (Figure 4). In addition, HECTIC performs slightly better than WAVE when the CR cache size gets larger. Thanks to the smart replacement policy, which stores the most important content items at each CN, PPCS provides a higher cache hit ratio than other strategies. PPCS then enhances the content diversity and cache utilization of limited-resource CS by pushing popular content items toward edge network caches while keeping the core network caches containing the low popular content chunks.
- **Impact of hop reduction ratio with different CR cache sizes**. For this evaluation, PPCS achieves the highest reduction ratio since it can keep content items closest to the user and the most recent and important objects can be stored at the edge CRs to avoid duplicated caches of chunks. While PPCS gains the highest QoE (Quality of Experience) with the lowest hop count by shifting the caches of popular content objects to the edge side, LCE once again performs worst since it treats and caches both popular and unpopular content objects in the same way by storing whole data at every CN along the retrieval path. In addition, as shown in Figure 5, though the CR cache size range is kept the same as the previous evaluation on the cache hit ratio, we realize that WAVE performs worse than ProbCache in the hop reduction ratio due to edge CNs’ underutilization tendency in WAVE. HECTIC also performs well but PPCS realizes the fastest response time of the content to the end user by minimizing the number of packets transmitted to higher level CRs and CP through a dynamic data processing mechanism utilizing the proposed collaborative edge-based caching scheme. Particularly, given that the earlier chunks are more popular than the later ones, by caching the initial chunks of the content, PPCS pushes content closer to the user and effectively diminishes the latency for content accesses.
- **Impact of the cache diversity enhanced ratio over various Alpha values** (compared to LCE with LRU). Cache diversity measures the total number of distinct content objects stored and the efficiency of cache replication and replacement in ICN. This network metric shows how well the caching mechanism can adapt to the content arrival with various content popularity levels. By employing the high-utility content chunks at content nodes to enhance cache diversity, HECTIC and PPCS outperform alternatives for effective content distributions in ICN interconnections (Figure 6). In addition, apart from HECTIC, which requires content internal popularity distribution (high complexity), we identify the threshold for the content arrival rate to the edge nodes and define the different caching policies for distinguished content types, with higher chunk priority given to the foremost chunks of each content. PPCS then avoids content redundancy by constraining a transmission path to store a maximum of one complete content object in the cache for maximizing the content diversity in ICN.

The evaluation results show that, in the case of other related caching strategies, a large amount of unnecessary cache space (CS) is used for storing the entire data of popular content items that are less likely to be asked again soon. In contrast, we propose PPCS in ICN nodes for storing a content progressively (from the edge side), which corresponds to its popularity and traffic load to improve the cache diversity of ICN interconnections. The popularity-aware caching scheme then efficiently utilizes the limited and valuable cache resource for deserved-to-be cached content objects to reduce the number of packets transmitted to higher level CRs and CP. In this way, we exploit the edge computing to resolve the cache redundancy issue in ICN so that the users can get the content faster, typically from the edge nodes via the enhanced content diversity for efficient content accesses. In addition, by mapping the content caching ratio at each CN with the content request arrival rate, i.e., the content popularity. Pushing content closer to users, we show that PPCS can improve key network performance considerably, especially in the case of popular content and limited cache storage. This mechanism is transparent to the user to increase content diversity in ICN, and spreads highly popular and important content items closer to users through edge caches for eliminating redundant traffic and wasteful cache space.

Overall, for all the evaluated metrics, PPCS consistently outperforms all other alternative approaches and greatly diminishes content cache redundancy through a wide range of content popularity distributions (Zipf) and CN cache sizes. Specifically, PPCS removes the tail chunks of unpopular content objects due to their negligible significance to reduce the size and number of unnecessary exchanged packets. Since the unpopular content with low utility can still be retrieved from the content producer, this does not result in degraded performance. The evaluation results then show that PPCS achieves the best performance over other related current caching schemes in minimizing network resource (cache storage) and cache redundancy to optimize cache utilization in ICN. The reason is that PPCS facilitates the efficient data transmissions via the edge layer and distributed processing among the on-path nodes in ICN. Given that an efficient caching scheme can reduce energy consumption by diminishing the traffic load via enhanced cache efficiency, as shown in our prior studies [4,40], the proposed popularity-aware caching scheme can be considered a scalable and feasible ICN caching approach for the FI implementation.

## 6. Conclusion and Future Work

Since ICN will play a vital role in the development of future networks as defined in Reference [7], in this article, we propose PPCS to realize a potential scalable and intelligent caching scheme. This novel caching mechanism eliminates cache redundancy in ICN and significantly improves the caching performance over alternative caching schemes.

By considering the sequential correlation among requests for chunks of a particular content, PPCS efficiently allocates content chunks at suitable CNs along the delivery path via a lightweight dynamic, collaborative caching scheme in sequential order. In addition, the combined caching placement and eviction policies of PPCS make (important) content objects as close as possible to users together with storing higher utility and more popular content chunks longer in CS and closer to the edge side. PPCS then offers optimal usage of the CN cache storage to avoid unnecessary content duplicates and achieves overall higher network performance compared to relevant alternatives (especially in the case of small-size CS). Particularly, by enhancing the content diversity in ICN and spreading highly popular content items close to users through edge caches, the proposal efficiently eliminates redundant traffic, decreases backbone traffic, latency, and potential operating power over hierarchical ICN interconnections.

As a result, PPCS with lightweight implicit collaborations lays down the foundation for the practical design of a ubiquitous and transparent ICN infrastructure for real-world networks to overcome the deficiency of current Internet architecture for users’ huge content demands, given that ICN is still in the developmental stage. This proposal then motivates fast, potential, and feasible ICN migration path for FI, which matches the goal of sustainability for FI content accesses, as defined in the global development agenda in 2030 of the United Nations [41].

For our future study, the realization of the scheme under larger scalability, distinct kinds of content services with different content sizes, and general ICN topology are needed to further estimate proposal efficiency. To make the proposal as realistic as possible for potential adoption in future networks, we will consider storing a failover copy of a content item in an appropriate CN in order to ensure content availability in case of link failure. Additionally, we will investigate the optimal number of replicas for each content based on caching cost, content size, and potential revenue from caching for maximized benefits. We also plan to extend the idea of PPCS for sensor-enabled ICN system with data sensing and further evaluate its energy-efficiency performance through the energy models as our prior studies in the context of the Green ICN system for the realization of the efficient green data collection, processing, and dissemination in ICN. Another unique approach is analyzing the accumulated popularity of same-category content items in which the objects that share the same attributes are grouped into a cluster and then verifying the benefit of PPCS over this method to enhance the overall potential gains from caching in ICN with regard to complexity and latency. 

## Figures and Tables

**Table 1:** The key simulation parameters.

Parameter	Value
Network topology	A complete 5-level ternary tree
Content size	400 MB
Number of objects	100,000
Payload size (chunk size)	1024 Byte
Link capacity among CNs	1 Gbps
Link capacity between user and CNs	100 Mbps
*CS* size	10^−4^ to 10^−3^ of all available content
Content distribution model	Zipf (α = 0.8)
*m* (exponential coefficient for content chunk caching)	2
*c_t_* (content access threshold)	the least access number of the top 30% most accessed content in a period
Runtime	700 s
